# Data Mining of Acupoint Characteristics from the Classical Medical Text: *DongUiBoGam* of Korean Medicine

**DOI:** 10.1155/2014/329563

**Published:** 2014-12-09

**Authors:** Taehyung Lee, Won-Mo Jung, In-Seon Lee, Ye-Seul Lee, Hyejung Lee, Hi-Joon Park, Namil Kim, Younbyoung Chae

**Affiliations:** ^1^Acupuncture and Meridian Science Research Center, College of Korean Medicine, Kyung Hee University, 1 Hoegi-dong, Dongdaemun-gu, Seoul 130-701, Republic of Korea; ^2^Department of Medical History, College of Korean Medicine, Kyung Hee University, 1 Hoegi-dong, Dongdaemun-gu, Seoul 130-701, Republic of Korea

## Abstract

Throughout the history of East Asian medicine, different kinds of acupuncture treatment experiences have been accumulated in classical medical texts. Reexamining knowledge from classical medical texts is expected to provide meaningful information that could be utilized in current medical practices. In this study, we used data mining methods to analyze the association between acupoints and patterns of disorder with the classical medical book* DongUiBoGam *of Korean medicine. Using the term frequency-inverse document frequency (tf-idf) method, we quantified the significance of acupoints to its targeting patterns and, conversely, the significance of patterns to acupoints. Through these processes, we extracted characteristics of each acupoint based on its treating patterns. We also drew practical information for selecting acupoints on certain patterns according to their association. Data analysis on* DongUiBoGam*'s acupuncture treatment gave us an insight into the main idea of* DongUiBoGam*. We strongly believe that our approach can provide a novel understanding of unknown characteristics of acupoint and pattern identification from the classical medical text using data mining methods.

## 1. Introduction

Throughout the history of East Asian medicine, different kinds of acupuncture treatment experiences have been accumulated in classical medical texts. In Korea, the medical book* DongUiBoGam* is considered as the representative of Korean medicine. This classic medical publication was completed in 1610 and published in 1613 under the Joseon Dynasty by a royal physician Heo Jun (1539–1615). It includes latest medical texts published in East Asia until then [1] and constitutes unique medical perspective on understanding the human body and treating disorders. Because of its significant value in medical aspects in East Asia, it became registered in UNESCO's Memory of the World Program in 2009.

In the field of East Asian medicine, various treatment experiences collected over time gradually formulated certain medical theories. Such medical theories inversely synthesized a series of treatment processes including diagnosis, pattern identification, and prescription. Pattern identification is a method of thinking which provides evidence for treatment by synthesizing and analyzing clinical data and differentiating patterns [2]. In East Asian medicine, methods of diagnosis and diagnostic classifications usually have preceded to identify the pattern of a certain disorder before actual treatment [3]. Traditional ways of diagnosis and diagnostic classifications were widely shared among physicians throughout the history of East Asian countries. However, each physician's detailed interpretations on the diagnostic information for pattern identification were not necessarily identical. Since pattern identification closely related to the physician's academic backgrounds, if one physician has different academic perspective from the others, the way of pattern identification could be varied. Therefore, it is necessary to investigate each way of pattern identification to figure out how it worked in the actual practice.

The publication of* DongUiBoGam* enabled Korea to develop unique acupuncture treatment theories [4]. Generally, acupoints and the target disorder can be related by the knowledge of the meridian system and its relevant organs [5, 6]. This process can be determined more in detail. When physicians treat patients with acupuncture, they can select the appropriate acupoints according to three basic principles: (1) local acupoints near the area where symptoms occur, (2) distant acupoints along the meridian, and (3) distant acupoints based on pattern identification [7, 8]. The first two principles of selecting acupoints are obvious if the knowledge on the meridian is understood. However, pattern identification, the key element of the third principle, is somewhat complex compared to the other principles. There are various ways of pattern identification by East Asian medical physicians according to their different academic backgrounds. In order to understand acupuncture treatments in* DongUiBoGam*, therefore, we need to study the way of pattern identification of this book.

To clarify the unique characteristics of East Asian medicine, several studies have attempted to reveal the relationship between treating methods and patterns using data mining and network science [9–13]. In one study, data mining algorithms were used to uncover useful patterns from various fields including classical East Asian medicine texts [14, 15]. Currently, developing a practical clinical decision support system for acupuncture therapy is required; it should make full use of modern methodologies of evidence-based medicine and utilize the abundant available information in current databases and data mining techniques [16]. Both research methodologies are providing new opportunities for understanding complex acupoint characteristics based on pattern identification [17]. However, systematic research on how to select acupoints based on pattern identification is still limited.

In this study, we analyzed and visualized the association between acupoints and patterns of disorders from the medical book* DongUiBoGam* using data mining methods. Before analyzing this association, we categorized various patterns of disorders into 25 patterns according to* DongUiBoGam*. Our results demonstrate how to acquire practical information for selecting acupoints based on pattern identification and help elucidate the characteristics of each acupoint regarding its relationship with components of pattern identification.

## 2. Methods

### 2.1. Data Source: Document

Since 1443, Joseon Dynasty, founded in 1392, took a strong leadership in collecting medical books published in East Asia and creating a compilation of the East Asian medical texts. The dynasty's purpose of this work was to restructure national medical system for the public [1]. Finally, Joseon Dynasty could complete the work on an extensive medical compilation* EuiBangYooChui* in 1445. With this precedent work on medical compilation, a medical book* DongUiBoGam* could be published in 1613 by using not only ancient medical classics such as* Huangdi Neijing* and* Shang Han Lun*, but also the latest medical texts until that time like* Yixuezhengchuan* (1515),* Yixuerumen* (1575), and* Wanbinghuichun* (1587) from China and* HyangYakJipSeongBang* (1443) and* EuiRimChwalYo* (1567) from Korea as reference.

Although* DongUiBoGam* cited a large number of Chinese medical texts, this book could be considered as the representative of Korean medicine because of its unique perspectives on understanding the human body and treating disorders. We can find these independent characteristics of* DongUiBoGam* with the preface of this book written in 1611 by Yi Jeonggu who was a Grand Master for Esteemed Merit, Minister of Personnel, Director of the Office of Special Advisors, and Director of the Office of Royal Decrees [18].

*Those of the medical profession often mention Huangdi and Qibo. Huangdi and Qibo studied the frames of the sky above and the natures of men below, and would not have thought it appropriate to record and describe medicine. Yet they shared questions and left records of complicated subjects that provided later generations with medical methods. This is how medical books have come to exist from our predecessors. From Changgong and Qin Yueren of antiquity to Liu Hejian, Zhang Zihe, Zhu Danxi, and Li Dongyuan of the recent past, countless schools have risen and theories have appeared, but the medical arts became more obscure and there were many cases that lost the original meanings of the Divine Pivot (靈樞). Many mediocre doctors disregard the fundamentals and arbitrarily practice medicine, running counter to the spirit of the classics, or lose the key substance by adhering to the old without versatility and straying with no criteria. This is why some kill instead when they are trying to save people.*

On the first paragraphs above, Yi introduced how medicine in East Asia has started from legendary Huangdi and Qibo and how this tradition of medicine has succeeded by remarkable physicians such as Liu Hejian, Zhang Zihe, Zhu Danxi, and Li Dongyuan after then. However, Yi did not think these medical traditions were always maintained in a good condition. On the contrary, he thought, medical tradition and the key substances of medicine became obscure and disregarded because of countless medical schools which have arisen so imprudently.

*Our king Seonjo (宣祖 1552–1608) grieved of the people's pain and came to be concerned with medicine, in extension of his benevolent wish to relieve the people with methods for curing the body. Thus in the year of 1596 he called the royal physician of the Imperial Medical Office, Heo Jun, and said, “Recent Chinese books on medicine are all trifle stuff that are merely copies and not worth reading. All medical books ought to be gathered into one book. People's diseases all come from the inappropriate care of their health, so the subjects about cultivation should come first, and the subjects on herbs and acupuncture should come later. Most medical books are too vast and complex, so only the essentials shall be selected. The reason that many die prematurely in the rural provinces and secluded streets without doctors and medicine is because although local herbs are produced abundantly in our country, people do not know of them. So we have sorted out and recorded the names of local herbs so that the people may more easily know about them.”*

Then Yi quoted King Seonjo's comment on these circumstances. King Seonjo wanted to change this unorganized situation and keep the medical fundamentals. So he called a royal physician Heo Jun and stressed three points what he thought essential in medicine: (1) gathering the essentials of all medical books into one book, (2) emphasis on cultivation of the body, and (3) emphasis on local herbs and acupuncture. King Seonjo expected him to complete a medical book based on his comments.

**Figure 1:**
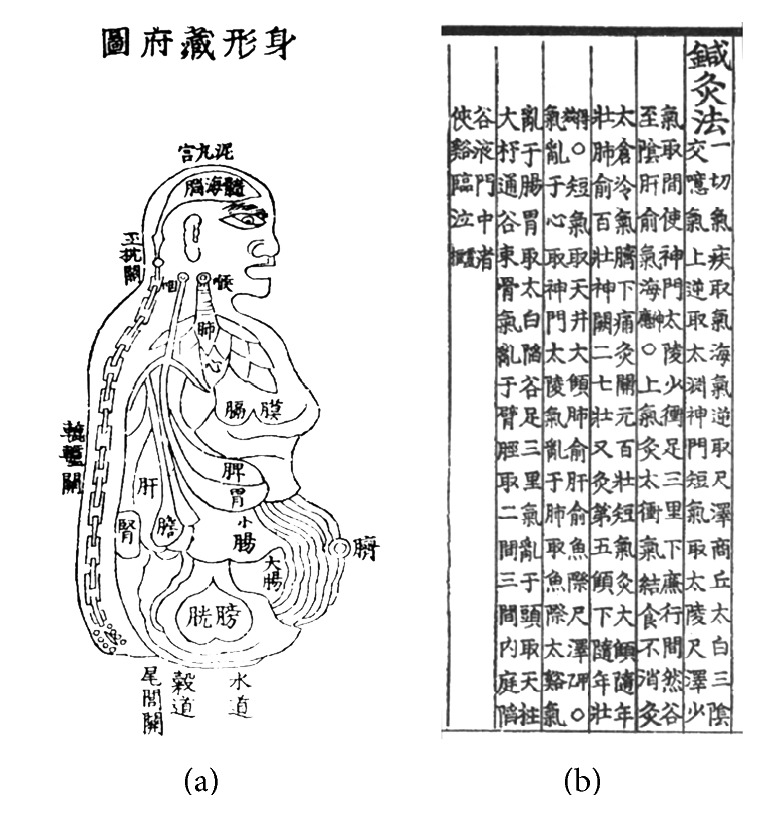
(a) The ShinHyungJangBuDo (chart of the overall body, viscera, and bowels) at the opening of the NaeGyeong chapter represents the medical perspective of* DongUiBoGam*. Heo Jun tried to express the common thread of this book of maintaining health through this picture; the circulation (“ascending of essence and qi” and “descending of fire”) of the body. Wiggling nose, opened mouth, and moving belly show the descending of “fire” through deep breath, and prominent spine and brain represent the ascending of “essence and qi” through meditation. (b) Acupuncture and Moxibustion Methods of subchapter “qi” in the NaeGyeong chapter of* DongUiBoGam*. The acupoint methods in subchapter “qi” contain the most frequently used acupoints: CV4, CV6, and ST36.

At the first page of* DongUiBoGam*, there is a picture called ShinHyungJangBuDo (Figure 1(a)). In this picture, we can grasp a basic idea on the cultivation of the body which emphasizes the circulation of the body and balance among the organs. Heo Jun tried to express the emphasis on cultivation in this picture with the idea of “descending of fire” via the cinnabar fields on the front side and “ascending of essence and qi” via the spine on the backside of the body through meditational breathing. Wiggling nose, opened mouth, and moving belly show the descending of “fire” through deep breath, and prominently described spine and brain represent the ascending of “essence and qi” through meditation.

The overall structure of* DongUiBoGam* is also closely related to Heo Jun's medical perspectives as well. It has a distinctive overall structure divided into five chapters: Internal Bodily Elements, External Bodily Elements, Miscellaneous Disorders, Herbs, and Acupuncture & Moxibustion. Unlike other medical texts that explain problematic disorders that originate from external causes most importantly, Heo tried to elucidate the basic structure of the human body first before discussing miscellaneous disorders in* DongUiBoGam*. Then, after explaining various Miscellaneous Disorders in detail, Heo introduced techniques of the treatment at the last chapters of Herbs and Acupuncture & Moxibustion.

### 2.2. Database Construction

The first chapter of* DongUiBoGam* is NaeGyeong, which addresses internal body elements [19]. There are 26 subchapters in NaeGyeong and 14 of them are titled “Acupuncture and Moxibustion Methods” (Figure 1(b)); these contain information on acupoints and disorders they treat. Using these sentences as the data source, we collected acupoint information and their target disorders as a dataset. Then we searched for the patterns of the target disorders from the corresponding subchapter of these disorders and categorized them into 25 patterns. Previously, we defined 25 representative patterns of disorder, which we then categorized into three pattern identification categories before compiling 25 patterns into a dataset [20].

To classify 25 patterns into three pattern identification categories, authors who are doctors of Korean medicine and majored in medical history and/or acupuncture discussed the structure of* DongUiBoGam*. The first pattern identification category consisted of five essential components of the human body: essence, qi, spirit, blood, and phlegm. The second pattern identification category concerned the five viscera and six bowels: liver, heart, pericardium, spleen, lung, kidney and gallbladder, stomach, small intestine, large intestine, bladder, and the so-called triple energizers (i.e., the upper, middle, and lower region of one's core, itself considered an organ in East Asian medicine). The third pattern identification category was composed of internal and external causes: food, overwork, sexual activity, wind, cold, dampness, dryness, and fire.

### 2.3. Data Mining and Network Analysis

From the data construction described above, we extracted data of cooccurrence frequencies and cooccurrence of acupoints from 25 patterns in three pattern identification categories. We extracted valuable information and knowledge from the data to understand the relationship between the acupoints and 25 patterns. In other words, we determined which specific patterns are more important and meaningful for an acupoint and which acupoints are more related to a pattern [21].

To accomplish this, we applied a “term frequency-inverse document frequency” (tf-idf) term weighting scheme to the cooccurrence table. tf-idf is a widely used term weighting scheme in the data mining field, especially in information retrieval systems because it quantifies the significance of terms in a particular document [22]. We also quantified the significance of acupoints to a pattern or of patterns to an acupoint. In the tf-idf scheme, term frequency tf_(*t*,*d*)_ is the number of times that term *t* occurs in document *d*; therefore tf_(*t*,*d*)_ represents how relevant the term *t* is to document *d*. Document frequency df_*t*_ is the number of documents that contain term *t*, showing how rare the term is in the system composed of documents. Rare terms are more informative than frequent terms; thus the inverse document frequency of *t* (idf_*t*_) is positively related to the informativeness of *t*. Arithmetically, idf is defined as log⁡⁡(*N*/df_*t*_) instead of *N*/df_*t*_ to diminish the effect of idf, where *N* is the number of whole documents [23]. Here, we calculated two types of tf-idf: tf-idf_(*p*,*a*)_ and tf-idf_(*a*,*p*)_, with the former defined by assigning a pattern to a term and an acupoint to a document, thus quantifying the significance of patterns to acupoints. Based on tf-idf_(*p*,*a*)_ values, each acupoint was represented by a vector of tf-idf weights in 25 dimensional vector spaces (25 is the number of patterns). To compare acupoints in the vector space, the tf-idf weights of each acupoint were normalized for the same length by the cosine normalization ($1/\sqrt{w_{1}^{2}+w_{2}^{2}+w_{3}^{2}\cdots +w_{M}^{2}}$) [2]. In the case of tf-idf_(*a*,*p*)_, all were calculated in the other way around. Therefore, using tf-idf_(*a*,*p*)_ values, each pattern id was represented as a vector in 114 dimensional vector spaces (the number of acupoints).

For further analysis, the relationship among acupoints was represented as a network. We defined a linkage when two acupoints were closer than 0.1 correlation distance (the threshold is defined by considering the distribution of correlation distances between every acupoint; the lower 1 percentile of the distribution is below 0.1 correlation distance). In the network, an average vector of each separated module was presented in a radar chart.

## 3. Results

### 3.1. Acupoint Characteristics

A total of 114 acupoints (341 before deduplication) were used to treat 114 different cases in the NaeGyeong chapter. We could extract 500 patterns (1082 before deduplication) which explain pathophysiological mechanisms from these 114 cases. Among 114 acupoints, 43 acupoints, which appeared more than three times in the text, were selected for the analysis, and 500 patterns were classified into 25 patterns with the three pattern identification categories before tf-idf analysis.

**Figure 2:**
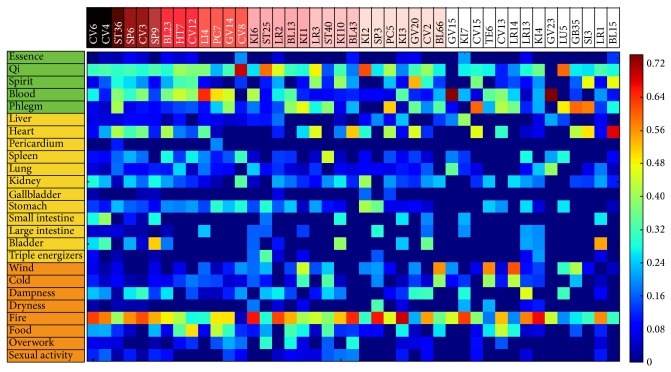
The tf-idf_(*p*,*a*)_ matrix. In this matrix, each acupoint (sample) is represented by a vector of tf-idf_(*p*,*a*)_ weights of 25 patterns in the three pattern identification categories (feature). To compare acupoints in the vector space, the tf-idf weights of each acupoint were normalized for the same length by the cosine normalization ($1/\sqrt{w_{1}^{2}+w_{2}^{2}+w_{3}^{2}\cdots +w_{M}^{2}}$). The 43 acupoints are presented on the* x*-axis of the array. The most frequently used acupoints were CV4 and CV6 (16 times each), ST36 (13 times), and SP6 and CV3 (12 times each). On the* y*-axis in Figure 2, 25 patterns are presented. The three pattern identification categories are shown in different colors on the* y*-axis (green: five essential components of the human body; yellow: viscera and bowels; orange: internal damage and external contractions).

The 43 acupoints are presented on the* x*-axis of the array in Figure 2. The most frequently used acupoints were CV4 and CV6 (16 times each), ST36 (13 times), and SP6 and CV3 (12 times each). On the* y*-axis in Figure 2, 25 patterns are presented. The three pattern identification categories are shown in different colors on the* y*-axis (green: five essential components of the human body; yellow: viscera and bowels; orange: internal damage and external contractions).

The five most valued tf-idf patterns among the five most frequently used acupoints are shown in Table 1. The five most frequently used acupoints were characterized from 25 patterns. The highest tf-idf values of the most frequently used acupoints were CV4 and CV6. Both acupoints similarly had greater tf-idf values in patterns such as “fire” and “qi,” except for CV6 which had relatively higher value in “small intestine” than CV4. Moreover, SP6 and CV3 also showed similar tf-idf values among the top five patterns. Both had greater tf-idf values in patterns such as “fire” and “heart,” except CV3 had higher value in “blood” than “qi,” which was the opposite from case of SP6. ST36 showed a relatively wide range of associations with the top five patterns. There were no significant differences among the top five patterns: “heart” (tf-idf: 0.42), “phlegm” (tf-idf: 0.41), “fire” (tf-idf: 0.40), “blood” (tf-idf: 0.38), and “qi” (tf-idf: 0.31). According to this data, we can say ST36 encompasses various disorders related to the patterns.

### 3.2. Network Analysis of Similar Acupoint Characteristics

**Figure 3:**
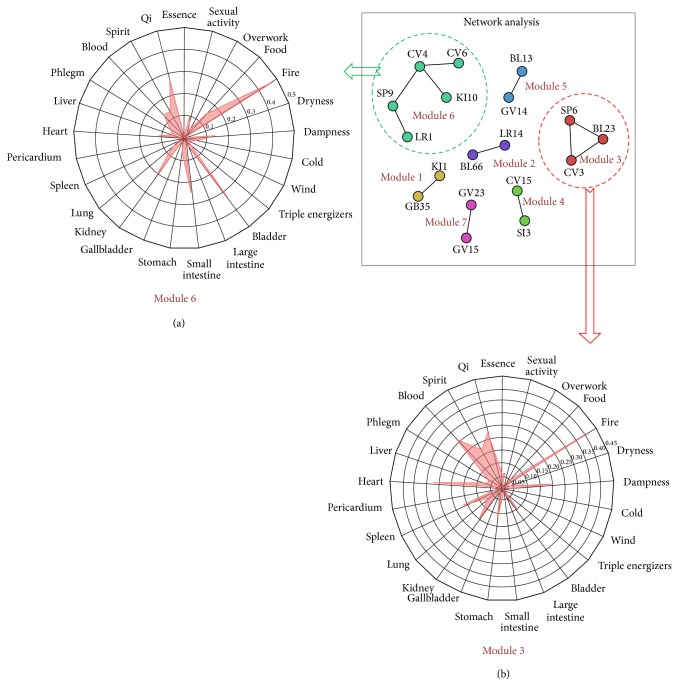
A network model of acupoints defined by tf-idf values with 25 patterns in the three pattern identification categories. (a) Radar charts showing characteristics of module number 6 (acupoints CV4, SP9, CV6, LR1, and KI10) in the network model. (b) Radar charts showing characteristics of module number 3 (acupoints CV3, SP6, and BL23) in the network model. Based on the charts, the characteristics of each module's acupoints can be understood. Acupoints CV6 and CV4 were both in the number six module. Acupoint CV4 acts as a hub among the other acupoints (SP9, CV6, and KI10). When evaluating the number six module, acupoint CV4 appears to play a key role. Acupoint CV4, with other acupoints in the number six module, has high values in the patterns “fire,” “small intestine,” “qi,” “bladder,” and “kidney.” On the other hand, acupoints CV3 and SP6 were in the number three module.

The characteristics of 43 acupoints were expressed by tf-idf values in 25 patterns. To detect acupoints which have similar characteristics, acupoints with less than 0.1 correlation distance among themselves were selected (Figure 3). Seven modules remained after this process.

The two module characteristics are shown on the radar charts (Figure 3). Based on the charts, the characteristics of each module's acupoints can be understood. Acupoints CV4 and CV6 are both in module 6 (Figure 3(a)). We can find acupoint CV4 acts as a hub among the other acupoints in that module (CV6, KI10, SP9, and LR1). When evaluating module 6, acupoint CV4 plays a key role. In addition, acupoint CV4 shares the top five patterns as “fire,” “small intestine,” “qi,” “bladder,” and “kidney” with other acupoints in module 6. On the other hand, acupoints CV3 and SP6 are in module 3 (Figure 3(b)). In module 3, acupoints CV3 and SP6 play a similar role in acupuncture treatment considering their highly associated patterns such as “fire,” “qi,” and “blood.”

### 3.3. Applicable Acupoints on Each Pattern of Disorders

**Figure 4:**
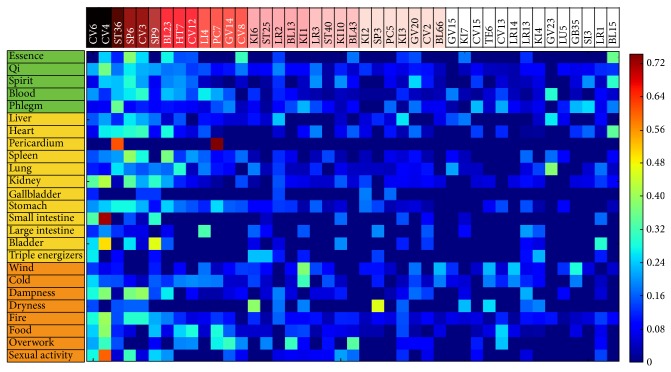
The tf-idf_(*a*,*p*)_ matrix. In the case of tf-idf_(*a*,*p*)_, all were calculated in the other way around. Using tf-idf_(*a*,*p*)_ values, each pattern id was represented as a vector in 114 dimensional vector spaces (the number of acupoints). In this matrix, each pattern (sample) is represented by a vector of tf-idf_(*a*,*p*)_ weights of 43 acupoints (feature). The most frequently used patterns in the NaeGyeong chapter of* DongUiBoGam* were “fire” (94 times), “qi” (89 times), “blood” (46 times), “phlegm” (39 times), and “heart” (34 times). Regarding “fire,” CV4 (tf-idf: 0.42), SP6 (tf-idf: 0.31), CV3 (tf-idf: 0.31), CV6 (tf-idf: 0.30), and SP9 (tf-idf: 0.23) were analyzed as applicable acupoints. Analysis of the pattern “qi” showed that acupoints CV4 (tf-idf: 0.38), SP6 (tf-idf: 0.28), CV6 (tf-idf: 0.23), CV3 (tf-idf: 0.23), and SP9 (tf-idf: 0.22) had high tf-idf values. Notably, the top two patterns, “fire” and “qi,” shared the same top five acupoints.

We also extracted tf-idf values based on 25 patterns in the three pattern identification categories (Figure 4). In Figure 3, we defined the characteristics of each acupoint with its association with patterns. In reverse, in Figure 4, we intended to find the most applicable acupuncture treatment options to a certain pattern. Five applicable acupoints are aligned with their targeting pattern in Table 2. The most frequently used patterns in the NaeGyeong chapter of* DongUiBoGam* were “fire” (94 times), “qi” (89 times), “blood” (46 times), “phlegm” (39 times), and “heart” (34 times).

Regarding the pattern “fire,” CV4 (tf-idf: 0.42), SP6 (tf-idf: 0.31), CV3 (tf-idf: 0.31), CV6 (tf-idf: 0.30), and SP9 (tf-idf: 0.23) were analyzed as applicable acupoints. Analysis of the pattern “qi” showed that acupoints CV4 (tf-idf: 0.38), SP6 (tf-idf: 0.28), CV6 (tf-idf: 0.23), CV3 (tf-idf: 0.23), and SP9 (tf-idf: 0.22) had high tf-idf values. Notably, the top two patterns, “fire” and “qi,” shared the same top five acupoints. Furthermore, except for the pattern “phlegm,” the other top four patterns all included acupoints located in the lower abdomen such as CV3, CV4, and CV6.

## 4. Discussion

A great number of treatment experiences have been accumulated through the long history of East Asian medicine and have been passed down through diverse medical texts. Accumulated treatment experiences which have clinical implications formed specific patterns, and these patterns inversely constituted evidence for the best treatment to patients. Patterns became associated with treating methods like herbs, acupuncture, and moxibustion. In case of acupuncture, a large number of acupoints were related to its treating patterns, and such related patterns constituted the characteristics of these acupoints. However, the patterns determined here have shown differences according to different treatment experiences or different academic backgrounds among physicians. Indeed, there have existed numerous trials for the most appropriate treatment to patients throughout the history of East Asian medicine. Therefore, we need to figure out how these patterns were formulated and associated with treating methods in each context of medical texts if we are willing to understand and utilize the medical experiences in current medical practices.

When publishing* DongUiBoGam*, as we have already seen in its own preface above, Heo Jun tried to refer to all medical books. Not only medical classics but also the latest medical texts current to that period were used as reference in this medical book. Nevertheless, not simply following the way of precedent medical texts,* DongUiBoGam* succeeded in making its own medical tradition with unique medical perspective on understanding the human body and treating disorders. As King Seonjo wished cultivation to be regarded importantly in* DongUiBoGam*, the royal physician Heo Jun tried to structure this medical book with the emphasis on understanding the human body first before discussing miscellaneous disorders. In this study, we analyzed and visualized the association between acupoints and patterns of disorders with the medical book* DongUiBoGam*. This medical book's patterns of disorders could be defined as 25 patterns in the three pattern identification categories according to the structure of* DongUiBoGam*. First of all, we applied data mining and network analysis methods to determine the characteristics of acupoints based on acupoints' association with 25 patterns. Secondly, to find categories of acupoints which share the common characteristics, we represented their relationship as a network and presented their shared characteristics on radar charts. Thirdly, we also analyzed each pattern's association with acupoints. With this process, we intended to select the most applicable acupoints on a certain pattern of disorders.

After analyzing the association between acupoints and 25 patterns using the tf-idf method, the characteristics of the 114 acupoints used in the NaeGyeong chapter of* DongUiBoGam* could be understood more specifically. For example, the most frequently used acupoints such as CV4 and CV6 shared patterns such as “fire” and “qi” with greater tf-idf values. But, in detail, CV4 had higher association with “small intestine” while CV6 and CV6 had higher relationship with “kidney” than CV4. Also, we could find considerable differences between CV4 and ST36. Contrary to CV4's association with the top five patterns which concentrated on the top two, ST36's association with the top five patterns was comparably equal among them. Through this analysis, we can assume that ST36's treating range is wider than CV4.

We identified 43 acupoints in the network with tf-idf values in 25 patterns, from which these acupoints in the similar position within the network were considered to have similar characteristics and were linked. We defined a linkage when two acupoints were closer than 0.1 correlation distance and then considered the connection of acupoints as a module. And then, from each module, the shared mean acupoint tf-idf values were plotted on radar charts. Through radar charts, relationships among 25 patterns were visually expressed. For example, we showed that CV4 and CV6 were in the same category on the network. Furthermore, in this network, we also found that the other acupoints like KI10, SP9, and LR1 share similar characteristics with CV4 and CV6.

With analyzed characteristics of acupoints, physicians can more easily select appropriate acupoints when they practice acupuncture considering pattern identification. For instance, when a physician treats patients with a headache, the headache can be divided into different patterns such as “fire” and “phlegm.” Different patterns lead to a different selection of acupoints. For the headache with the pattern “fire,” CV4, which shows the strongest association with it among acupoints, can be considered. And, for the headache with the pattern “phlegm,” ST36 which has the highest association with “phlegm” can be considered for acupuncture treatment.

In the NaeGyeong chapter of* DongUiBoGam*, we could find that acupoints located in the lower abdomen such as CV3, CV4, and CV6 were more frequently used than other acupoints. And patterns such as “fire” and “qi” were the top two patterns in the NaeGyeong chapter of* DongUiBoGam*. Moreover, by comparing the association of acupoints with the patterns and vice versa, we could also determine that there is an intimate relationship between the acupoints in the lower abdomen and the patterns “fire” and “qi.” This intimate relationship between the lower abdomen and “fire” and “qi” reminds us of the picture we saw in the first section of this paper, the ShinHyungJangBuDo (Figure 1(a)). The main idea of the ShinHyungJangBuDo was the circulation of the body and the balance among the organs. And, for the circulation, each person needs to “descend the fire” and “ascend the essence and the qi” in the body through meditational breathing. On the other side, the original name of CV4 and CV6 can be translated as “cinnabar field” and “sea of qi.” And both acupoints were thought of as “the basis of the five viscera and six bowels, the root of twelve meridians, the door to breathing, and the actual source of life qi” in* DongUiBoGam*. Through this data analysis on acupuncture treatment in* DongUiBoGam* and comparing it with the important points of the ShinHyungJangBuDo, we could get an insight into the main idea of* DongUiBoGam* which was stressed by King Seonjo and the author of this book Heo Jun.

This study has several limitations. First, the 25 patterns and the three pattern identification categories may not be sufficient to embrace all of the information in* DongUiBoGam*. Some information such as yin/yang or deficiency/excess could not be included in the 25 patterns because of their changing meanings according to different situation and context. Secondly, by simplifying numerous causes of disorders into 25 patterns and the three pattern identification categories, some information could have been missed. For example, in the case of the pattern “fire,” there were various types of fire such as sovereign fire, ministerial fire, and heat. However, since all of these fire-related terms were simplified into the pattern “fire,” we could not consider detailed implications of various related terms. Thirdly, the number of cases was insufficient to definitively describe the characteristics of 114 acupoints in the NaeGyeong chapter of* DongUiBoGam*. Only 43 acupoints among 114 were used more than three times in the NaeGyeong chapter, whereas 54 acupoints were used just once. Therefore, to obtain more detailed characteristics of each acupoint, analysis with more data sources from various medical texts and comparison of the results among them are required.

Currently, the clinical value of traditional East Asian medical practices has been highly under debate [24–26]. However, empirical medical records have been accumulated in traditional medical texts throughout the history. Therefore, studying traditional medical texts and reexamining the records may provide meaningful information for current medical practices. In East Asian tradition, various medical texts have their own unique system according to the author's perspective of the human body and disorders. Therefore, to understand the system in each medical text more accurately, each medical text should be studied with respect to its own system first and then compared to others in terms of similarities and differences. A thorough understanding of various medical texts can help physicians treat patients more effectively. We strongly believe that our data mining approach represents a new method for understanding the characteristics of acupoints.

## Figures and Tables

**Table 1:** The five most frequently used acupoints with the five most valued tf-idf patterns.

	Patterns				
Acupoints	(tf-idf)				
(frequency)	1st	2nd	3rd	4th	5th
	(tf-idf)	(tf-idf)	(tf-idf)	(tf-idf)	(tf-idf)
CV4	Fire	Small intestine	Qi	Bladder	Kidney
(16)	(0.58)	(0.40)	(0.33)	(0.32)	(0.23)

CV6	Fire	Qi	Kidney	Small intestine	Blood
(16)	(0.63)	(0.31)	(0.29)	(0.28)	(0.27)

ST36	Heart	Phlegm	Fire	Blood	Qi
(13)	(0.42)	(0.41)	(0.40)	(0.38)	(0.31)

SP6	Fire	Qi	Heart	Blood	Dampness
(12)	(0.56)	(0.32)	(0.32)	(0.31)	(0.27)

CV3	Fire	Blood	Heart	Dampness	Qi
(12)	(0.61)	(0.38)	(0.37)	(0.31)	(0.29)

**Table 2:** The five most frequently used patterns with the five most valued tf-idf acupoints.

	Acupoints				
Patterns	(tf-idf)				
(frequency)	1st	2nd	3rd	4th	5th
	(tf-idf)	(tf-idf)	(tf-idf)	(tf-idf)	(tf-idf)
Fire	CV4	SP6	CV3	CV6	SP9
(94)	(0.42)	(0.31)	(0.31)	(0.30)	(0.23)

Qi	CV4	SP6	CV6	CV3	SP9
(89)	(0.38)	(0.28)	(0.23)	(0.23)	(0.22)

Blood	CV3	GV23	LI4	SP6	BL23
(46)	(0.31)	(0.31)	(0.28)	(0.28)	(0.24)

Phlegm	ST36	SI3	CV15	GB35	KI1
(39)	(0.37)	(0.26)	(0.24)	(0.24)	(0.23)

Heart	BL15	CV3	SP6	BL23	ST36
(34)	(0.36)	(0.32)	(0.31)	(0.30)	(0.28)
